# Epidemiology, Clinical Significance, and Diagnosis of Respiratory Viruses and Their Co-Infections in the Post-COVID Era

**DOI:** 10.3390/pathogens14030262

**Published:** 2025-03-07

**Authors:** Kaia M. Contes, Benjamin M. Liu

**Affiliations:** 1 Department of Biology, Howard University, Washington, DC 20059, USA; kaia.contes@bison.howard.edu; 2 Division of Pathology and Laboratory Medicine, Children’s National Hospital, Washington, DC 20010, USA; 3 Department of Pediatrics, George Washington University School of Medicine and Health Sciences, Washington, DC 20010, USA; 4 Department of Pathology, George Washington University School of Medicine and Health Sciences, Washington, DC 20037, USA; 5 Department of Microbiology, Immunology & Tropical Medicine, George Washington University School of Medicine and Health Sciences, Washington, DC 20037, USA; 6 Children’s National Research Institute, Washington, DC 20012, USA; 7 The District of Columbia Center for AIDS Research, Washington, DC 20052, USA

**Keywords:** SARS-CoV-2, respiratory virus, influenza virus, respiratory syncytial virus, enterovirus, multiplex molecular testing, PCR, epidemiology, diagnosis

## Abstract

Severe acute respiratory syndrome coronavirus 2 (SARS-CoV-2), a novel human coronavirus, emerged in late 2019 and rapidly evolved into a pandemic around the world. The coronavirus disease (COVID-19) pandemic has dramatically changed the epidemiology and seasonality of other traditional respiratory viruses, e.g., influenza, respiratory syncytial virus, enterovirus, etc. These traditional respiratory viruses have transmission mode and clinical symptoms similar to SARS-CoV-2 but may differ in clinical outcomes and management. Co-infection between SARS-CoV-2 and one or more traditional respiratory viruses have been reported in the literature but have shown mixed evidence in clinical outcomes. With SARS-CoV-2 evolving into mild Omicron variants, it is believed that SARS-CoV-2 co-circulates with other respiratory viruses, which in turn affect the epidemiology and clinical course of respiratory viral infections. In response to these changes, multiplex molecular tests for SARS-CoV-2 and one or more traditional respiratory viruses are attracting more attention in the field and have been developed into a variety of testing modalities. In this review, we describe the seasonality (i.e., in the Northern Hemisphere), epidemiology, and clinical significance of traditional respiratory viruses and their co-infection with SARS-CoV-2 in the post-COVID era. Furthermore, we review commonly used multiplex molecular tests and their applications for the detection of respiratory viruses and their co-infections. Altogether, this review not only sheds light on the epidemiology and clinical significance of respiratory viral infections and co-infections in the post-COVID era, and but also provides insights into the laboratory-based diagnoses of respiratory viral infections using multiplex molecular testing.

## 1. Introduction

Severe acute respiratory syndrome coronavirus 2 (SARS-CoV-2) is a significant respiratory virus responsible for claiming over 6 million lives worldwide, inciting a global pandemic most known as coronavirus disease (COVID-19) [1,2,3,4,5]. It is believed that SARS-CoV-2 was first detected in Wuhan, China, in late 2019, and then kept evolving into different variants. SARS-CoV-2 falls within the coronavirus family of zoonotic viruses, which also includes the Middle Eastern Respiratory Virus (MERS-CoV), SARS-CoV, and other non-SARS-CoV-2 seasonal human CoVs (HCoVs) [6,7,8,9,10,11]. The high transmissibility and infection rates of SARS-CoV-2 set it apart from other coronavirus variants. While SARS-CoV is similarly an airborne virus, SARS-CoV-2 can be contracted and spread before the onset of symptoms and is spread just as frequently by asymptomatic individuals [12]. This challenges public health efforts, as it is harder to control the influx of symptoms when patients have different timelines regarding presymptomatic or asymptomatic infection. SARS-CoV-2 infection has varying adverse effects on infected individuals, ranging from mild to severe/critical. According to the World Health organization (WHO) [13], symptomatic patients typically experience common symptoms of fever, cough, fatigue, and loss of taste or smell, along with less common symptoms, such as skin irritation, bodily aches, sore throat, headache, diarrhea, and eye irritation [14]. Moreover, more severe illness is associated with serious breathing/lung impairment, which requires immediate attention. Some of the worst cases will experience life-threatening conditions, like acute respiratory distress syndrome, which requires intensive care. However, some individuals experience prolonged and severe symptoms following SARS-CoV-2 infection, known as long COVID [15]. This multisystemic condition has affected over 65 million people globally, increasing the risk for conditions such as type 2 diabetes, chronic fatigue, and cardiovascular disease [15].

**Figure 1:**
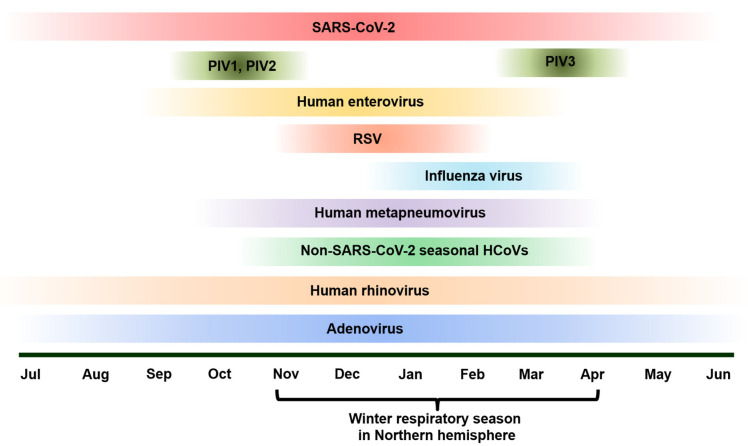
The seasonality of traditional respiratory viruses and SARS-CoV-2 in the Northen Hemisphere in the post-COVID era. While adenovirus and human rhinovirus circulate and cause respiratory infections year round, the time window between late fall and spring (between November and April) is believed to be the winter respiratory season in the Northen Hemisphere. In this timeframe, influenza viruses, RSV, and hMPV show elevated activities. Enteroviruses peak in activity in late fall. Although seasonal HCoV can cause mild colds year round, it has higher activity in the winter respiratory season. PIV1 and 2 are active in October and November, while PIV3 has high activity in March and April. SARS-CoV-2 co-circulates with the above-mentioned traditional respiratory viruses and causes infection year around.

SARS-CoV-2 spread rapidly worldwide starting in late 2019, but the rate of infection of many respiratory pathogens started to decline as most individuals followed major guidelines to slow the spread of the virus [12]. Since 2021, there have been changes in the epidemiology and the seasonality (for example, in the Northern Hemisphere) of pathogens such as influenza, respiratory syncytial virus (RSV), human metapneumovirus (hMPV), enterovirus, rhinovirus, parainfluenza virus (PIV) 1–4, and adenovirus [16,17,18]. In addition, with SARS-CoV-2 and respiratory viruses circulating simultaneously, co-infections between SARS-CoV-2 and respiratory viruses have become prevalent and have influenced patient outcomes [14,19] (Figure 1).

Before the pandemic, seasonal influenza typically peaked in the Northern Hemisphere in late winter, e.g., the first half of February, while the Southern Hemisphere saw peaks in late July. During the COVID-19 pandemic, seasonal influenza A and B viruses were still prevalent in many areas, causing unfavorable symptoms and complications even with readily available vaccines. The post-pandemic peak in the Northern Hemisphere occurred as early as December, with the duration of the epidemic increasing to 11 weeks. Although the post-pandemic peak in the Southern Hemisphere was not significantly changed, the influenza epidemics increased in length to 20 weeks [20]. As both influenza A/B and SARS-CoV-2 have similar transmission patterns (contact with aerosolized droplets and contaminated surfaces) and symptoms, and they typically circulate in seasonal outbreaks, they pose a significant public health threat due to the potential for co-infection [21].

Another appealing example is RSV, which is the leading cause of lower respiratory tract infections in young children, most prevalent in children under two years of age [22]. RSV is the second leading cause of infant deaths, making it a significant public health concern. Before the COVID-19 pandemic, in the Northern Hemisphere, RSV typically peaked in the winter season, around January, while in the Southern Hemisphere, the virus peaked in late July. After the COVID-19 pandemic, this pattern of RSV appeared 1–2 months early, and there was a larger gap in the Southern Hemisphere, which showed an earlier peak than usual during the first wave [20,23]. With the known contribution of RSV to increased respiratory distress and bronchiolitis in young children, the addition of SARS-CoV-2 infection can even further exacerbate the severity of RSV symptoms [24].

Notably, there are various important factors to be considered when examining the epidemiology of co-infections of respiratory viruses and their impact on clinical manifestations or outcomes. Respiratory viral co-infections have been reported at the cellular and animal experimental levels with the phenomenon of viral interference. These effects vary depending on the order of infection. From a clinical point of view, unlike in cellular model or animal model experiments, respiratory virus co-infections are also influenced by whether or not the viral epidemics involved occur simultaneously. At the clinical level, co-infection cannot occur if the epidemic periods are different. With SARS-CoV-2 evolving into mild Omicron variants and co-circulating with other respiratory viruses, there is a major knowledge gap regarding the impact of SARS-CoV-2 on the epidemiology and seasonality of other traditional respiratory viruses. Furthermore, the change in the virus circulation requires updated strategies in the timely detection of SARS-CoV-2 and other traditional respiratory viruses, e.g., influenza viruses, RSV, enteroviruses, human rhinoviruses, adenoviruses, PIVs, and seasonal HCoVs. There is an urgent need to implement multiplex molecular tests for SARS-CoV-2 and other traditional respiratory viruses, especially point-of-care (POC) tests. In this review, we assess the epidemiology and impact of SAR-CoV-2 co-infections on other respiratory viruses. Furthermore, we review multiplex molecular testing for the detection of respiratory viruses and co-infections with SARS-CoV-2. This review not only sheds light on the updated epidemiology of respiratory viruses in the post-COVID era but also provides insight into the appropriate application of multiplex molecular testing for the detection of respiratory viruses, especially POC testing.

## 2. Co-Infection of SARS-CoV-2 with Viral Respiratory Infections

Co-infections occur when a host body is infected by multiple pathogens simultaneously. As expected, this can complicate the symptoms and diagnoses of diseases. When co-infection is seen with respiratory pathogens, it is significant because a combination of symptoms may occur from multiple viruses, creating a destructive recipe for the body’s immune system, which may lead to prolonged illness and severe outcomes, especially in immunocompromised or immunodeficient patients [2,4,7,25,26]. At the cellular or molecular level, infections with other respiratory viruses, e.g., influenza A virus and RSV, might provide transient resistance to SARS-CoV-2, as the former viruses may trigger cellular responses, cytokine production, and innate immune responses that block the infection due to the latter viruses [27]. But when a patient’s immune system is weakened, a severe co-infection with different respiratory viruses may happen [2]. With the rise of the COVID-19 pandemic at the beginning of 2020, SARS-CoV-2 co-infections with other viral and bacterial pathogens sparked concern for the potential of exacerbating the already severe symptoms and outcomes that are associated with SARS-CoV-2 infection [25]. Evidence from a comprehensive analysis of more than 2 million global SARS-CoV-2 samples showed that these co-infections have occasional occurrence, making up around 0.35% of observed infections in individuals displaying both severe and mild symptoms [26]. While co-infection severity and frequency are continually being researched, there is growing evidence and understanding of how SARS-CoV-2 impacts various respiratory viruses and their diagnosis [26].

### 2.1. Influenza Viruses

Recent studies have shown that co-infection between SARS-CoV-2 and influenza strains has been associated with increased morbidity and mortality rates compared to individuals with single infections [28]. Influenza’s effect on SARS-CoV-2 hinders immune responses and reduces antibody levels. The unique abilities of influenza viruses and SARS-CoV-2 to modulate innate immunity may also lead to inflammatory responses and cytokine storms, which may complicate clinical courses [2,4,29,30,31,32]. Strikingly, patients who are co-infected have a mortality risk almost twice as high as those only infected with SARS-CoV-2 [21,28]. Vaccination for both SARS-CoV-2 and influenza provides an effective way for individuals to protect themselves against viruses and potential co-infection. Some studies have shown that influenza vaccines can cross-protect against SARS-CoV-2 and potentially mitigate the risk of severe risks, such as mortality and severe symptoms [28]. This can probably be explained by the concept of trained immunity, in which an unrelated vaccine may trigger an innate immune memory that can prevent infection due to another unrelated pathogen [33].

For the identification of co-infection between SARS-CoV-2 variants and influenza viruses, RT-PCR assay and whole genome sequencing were used, which allowed variants like Delta and Omicron to be differentiated, which is crucial for understanding individual strain epidemiology [34]. After performing these tests, statistical analyses were performed using logical regression to understand the associations between the SARS-CoV-2 variants and co-infections with influenza A. Using this model, the researchers were able to evaluate the impact of the co-infections on patient outcomes, such as symptoms, while taking patient demographic and medical history into account. This aided in understanding how co-infections with different strains may influence clinical outcomes. The study found a 33% co-infection rate among SARS-CoV-2-positive patients, with higher rates in those with the Delta variant. In-depth diagnostic testing is crucial for understanding viral co-infections. RT-PCR has been proven to be more effective than rapid antigen-based assays, though the latter are often more readily available [34].

### 2.2. RSV

While RSV is typically seasonal, with epidemics usually occurring in the winter months (Figure 1), there is plenty of opportunity for SARS-CoV-2 co-infection and co-circulation to transpire, as the two are similar in their transmission method (aerosolized droplets). Accordingly, as both can be prevented by wearing masks, isolating from infected individuals, and disinfecting communal surfaces, there was a drop in the reported RSV cases in the winter of 2021–2022, when the COVID-19 pandemic was still in full effect. However, once pandemic restrictions were lifted, RSV cases spiked, even in some cases higher than the pre-pandemic level [24]. This increased prevalence of RSV has increased the likelihood of SARS-CoV-2 and RSV co-infection, particularly in children, as they are most susceptible to respiratory viruses in general [35]. Children have also been found to be more susceptible to respiratory viral co-infections than adults overall [36]. While recent studies have shown that co-infection rates between RSV and SARS-CoV-2 are relatively sparse at 3%, their mere occurrence provides a significant understanding of how these viruses interact and their impact on patient outcomes [36].

As with many respiratory viruses, SARS-CoV-2 and RSV have similar symptoms, complicating the diagnostic process. Multiplex PCR tests are the most common laboratory tests used to determine the presence of multiple pathogens in a sample. Once the predominant viral pathogen is determined, it is typically treated as the primary focus while also treating the other virus, with RSV treatment being more focused on respiratory support. However, cases are mostly treated on an individualized basis, as increased severity may be seen in infected individuals who may need more intensive treatment, which is why understanding and detecting co-infections is essential [24].

### 2.3. hMPV

There is little significant understanding of the effect of hMPV on SARS-CoV-2 co-infection. Through chest computed tomography (CT) scanning and RT-PCR, a study in Iran in 2020 found three cases of SARS-CoV-2 and hMPV co-infection in young children (a 13-month-old and two six-year-olds). All three children expressed similar symptoms of fever, cough, and malaise, will all three passing away within 2 days of being hospitalized for their infections. The researchers speculated that hMPV’s direct effect on inflammation, interferon secretion patterns in the respiratory tract, and the causation of asthma may lead to increased SARS-CoV-2 susceptibility. The study emphasized the importance of testing for respiratory viral co-infections in young children, as they can potentially be fatal [37]. Furthermore, another study in 2020 detailed the case of a 57-year-old woman with pre-existing health conditions who tested positive for both hMPV and SARS-CoV-2 simultaneously (tested via respiratory pathogen panel (RPP) and RT-PCR). The patient expressed symptoms of dry cough and upper respiratory symptoms. SARS-CoV-2 and hMPV co-infections are infrequent in adults, and those with underlying or pre-existing health conditions may be at increased risk [38]. The knowledge out there indicates that these co-infections are typically uncommon, and the outcomes are not significantly different from singular SARS-CoV-2 infection. However, in young children and those with pre-existing health conditions, these co-infections may have more severe clinical outcomes and implications [37,38,39].

### 2.4. Enterovirus

Belonging to the family *Picornaviridae*, the genus *enterovirus* consists of enteroviruses, rhinoviruses, and polioviruses, which can be further divided into hundreds of subtypes [40,41,42]. Enteroviruses can cause a wide range of respiratory and neurological issues, sores, and rashes on the hands, mouth, and, in severe cases, encephalitis and acute flaccid paralysis [40]. When co-infected with SARS-CoV-2, enteroviruses can potentially produce severe clinical outcomes, and, as with most respiratory co-infections, there are higher potential risks of hospitalization and a need for inclusive diagnostic testing [39]. While many of the documented co-infections with SARS-CoV-2 and enterovirus are with rhinoviruses, there have been some studies that have documented non-rhinovirus enteroviruses. One study from Jordan focusing on upper respiratory tract infections (URTIs) found three cases of co-infections of SARS-CoV-2 and enterovirus co-infections and highlighted enteroviruses as the most common pathogen of viral co-infection [43]. The research highlighted that, generally, enteroviruses have a high tendency to co-infect with other viruses, including SARS-CoV-2, emphasizing the consistent need for multiplex testing methods to account for this possibility.

One review highlighted 130 studies where bacterial, fungal, and respiratory co-infections with SARS-CoV-2 were discovered in pediatric cases. Across the 130 studies predominantly using RT-PCR, only five cases of enterovirus co-infection with SARS-CoV-2 were found [44]. This may indicate that these co-infections are not as prevalent as others. However, one study found that culturing SARS-CoV-2, coxsackievirus A7 (CVA7), and enterovirus A71 (EV-A71) in Vero E6 and HEK293A cells produced an inhibitory effect on the SARS-CoV-2 virus. In addition, they found that hamsters co-infected with SARS-CoV-2 and enteroviruses exhibited non-severe clinical symptoms, even presenting with a smaller reduction in body weight and lower levels of SARS-CoV-2 in the lungs than those singularly infected with SARS-CoV-2 [45]. This study provides significant evidence that SARS-CoV-2 co-infections with enteroviruses may actually lessen the severity of SARS-CoV-2, providing critical evidence or understanding the interaction between the viruses. While the direct impact of SARS-CoV-2 co-infections with enteroviruses are still being studied, current animal and cell models provide promising evidence that enteroviruses may mitigate the effects of severe SARS-CoV-2 symptoms [45]. One hypothesis is that enteroviral infection may trigger interferon and interferon-stimulated genes mediated innate immune antiviral responses, which may play a role in restricting SARS-CoV-2 infection [46]. There is still a need for further research on the prevalence of these co-infections and how they affect different populations.

### 2.5. Rhinoviruses

A study in Southern Brazil assessing 20 respiratory pathogens during the COVID-19 pandemic uncovered significant findings about SARS-CoV-2 and rhinovirus co-infection in children between 2 and 18 years of age. The study found that out of 436 participants, 49.5% were infected with rhinovirus, making it the most detected pathogen. A total of 7.1% of the participants had co-infection with rhinovirus and SARS-CoV-2. Consequently, rhinovirus had a higher hospitalization rate when co-infection occurred with another virus, including SARS-CoV-2, while SARS-CoV-2 infection alone had a lower hospitalization rate [47]. Another study in 2020 in Brazil found that other than SARS-CoV-2, rhinovirus appeared to be the main virus circulating at the time. In their study, they found that rhinovirus was the only virus to co-occur with SARS-CoV-2, with 7 out of 91 patients experiencing SARS-CoV2 and rhinovirus co-infection [48].

A complication that arises in diagnosing COVID-19 with rhinovirus and SARS-CoV-2 is that they are expressed similarly regarding patient symptoms. For this reason, comprehensive testing is emphasized to account for any co-infections that may occur. This can be achieved through RT-PCR testing, which can detect multiple pathogens. Overall, recent studies that have explored co-infection between SARS-CoV-2 and rhinovirus have highlighted the importance of understanding SARS-CoV-2 infection and management in pediatric populations. While SARS-CoV-2 alone may only cause mild illness in children compared to adults, the presence of rhinoviruses may lead to more severe illness and hospitalization, which is a significant cause for concern [47].

### 2.6. PIV

One study used a multiplexed respiratory panel (RP) test, BioFire RP 2.1, which tests for a broad range of respiratory viruses at once using samples from nasopharyngeal swabs. The study found that there were no co-infections between PIV-1 and SARS-CoV-2. PIV-2+SARS-CoV-2 accounted for 0.89%, PIV-4+SARS-CoV-2 accounted for 0.48%, and PIV-3+SARS-CoV2 accounted for 1.77% [49]. While these percentages are relatively low, co-infections of PIV and SARS-CoV-2 are known to exacerbate symptoms and call for more supportive care than a singular infection might.

Furthermore, a recent case of co-infection was studied in a 73-year-old woman with a history of bilateral bronchiectasis [50]. She came to the hospital presenting with symptoms of fever, rhinitis, and increased dyspnea. Physicians ran a BioFire panel on her and discovered that she was co-infected with both SARS-CoV-2 and PIV-3. Accordingly, she was placed in the intensive care unit of the hospital and was treated for parainfluenza with injectable cefoperazone+sulbactam and oseltamivir, as well as steroids and low-molecular-weight heparin for high D dimer levels. Her health quickly turned around, and she was able to leave the hospital after eight days. This case emphasizes the need for the early detection of co-infections, as they can lead to more severe illnesses. In this case, the patient was able to return to good health swiftly because she was treated quickly [50]. Even though co-infections with various respiratory viruses are relatively infrequent, the importance of comprehensive viral testing is evident for understanding the effects and early detection in patients who may experience severe illness if not treated [50].

### 2.7. Adenovirus

Adenovirus circulates and causes respiratory infections year round (Figure 1). Studies have shown that only 5–7% of SARS-CoV-2 co-infections are adenovirus-related [51]. While co-infection between adenoviruses and SARS-CoV-2 is generally rare, it is still vital to consider adenoviruses when conducting diagnostic testing, as they could potentially lead to more severe outcomes. While adenoviruses account for only a small percentage of respiratory viral co-infections, when reported, they have typically been in individuals with underlying health conditions. One reported case in Colombia was a 40-year-old diabetic man whose diabetes was poorly taken care of. He experienced adverse symptoms from COVID-19, and it was discovered that he had a co-infection with adenovirus. This exemplifies why it is crucial to test for multiple pathogens, even when it is typically a rare occurrence [52]. It is possible that adenovirus and SARS-CoV-2 co-infection may cause adverse clinical outcomes in those with underlying health problems. It may require careful therapeutic management for those affected [52].

### 2.8. Non-SARS-CoV-2 Seasonal HCoVs

There are four species of non-SARS-CoV-2 HCoVs that are endemic and exhibit seasonal circulation in winter months in the Northern Hemisphere (Figure 1), including two Alpha-coronaviruses (HCoV-NL63 and HCoV-229E) and two Embecoviruses (HCoV-OC43 and HCoV-HKU1) [9]. Non-SARS-CoV-2 HCoVs are typically responsible for mild respiratory illness but can cause severe respiratory presentations, particularly in immunocompromised individuals [53]. Wilson and colleagues recently conducted a systematic review on the global epidemiology and seasonality of seasonal human CoVs before and during the COVID-19 pandemic [53]. There was a substantially lower prevalence of HCoVs in 2020 compared to previous years, suggesting that the COVID-19 pandemic has had an impact on the epidemiology of non-SARS-CoV-2 HCoVs [53].

Recent hCoV infections may be associated with a reduced severity of COVID-19, but they have nothing to do with the decreased number of SARS-CoV-2 infections [54]. Antibody levels to seasonal human CoVs and SARS-CoV-2 were determined at the onset of COVID-19 pandemic [55]. Anderson et al. found that ∼20% of these individuals had non-neutralizing antibodies against seasonal human CoVs, which can cross-react with SARS-CoV-2 spike and nucleocapsid proteins [55]. These antibodies against seasonal human CoVs were boosted following SARS-CoV-2 infection, but they may not lead to protection against SARS-CoV-2 infections or hospitalizations [55]. There is still scarce information about co-infection detected by molecular methods.

## 3. Multiplex Molecular Testing for Respiratory Viruses and Their Co-Infections

**Figure 2:**
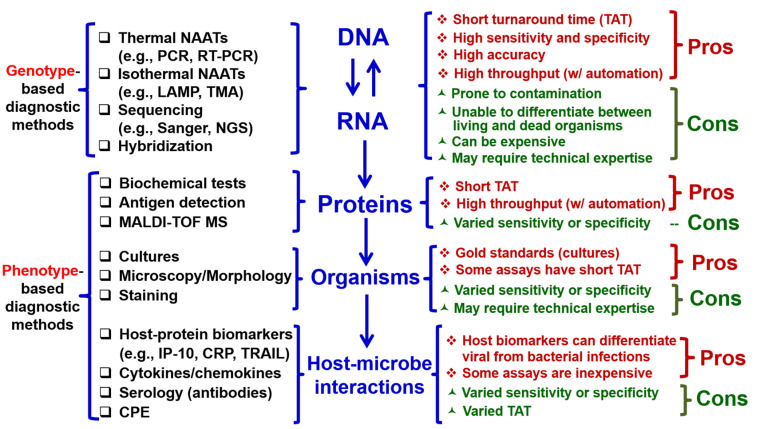
Classification of clinical microbiological testing methods for infectious disease diagnostics and comparison of their pros and cons. (**Middle**) The central dogma of molecular biology reflects the basis of the biological processes of a living microorganism, from transcription (DNA to RNA) and reverse transcription (RNA to DNA) to protein synthesis (RNA to proteins). In a disease state, host–microbe interactions, e.g., the generation of host–protein biomarkers (e.g., IP-10, CRP), cytokines/chemokines, and antibodies, play important roles in the host responses and pathogenesis of infectious diseases. (**Left**) Based on the nature of the targets of detection, clinical microbiologic methods for infectious diseases diagnostics can be divided into four groups, i.e., those detecting nucleic acids (DNA/RNA), proteins, organisms, or host–microbe interactions. First, the genetic materials of microorganisms, e.g., DNA and RNA, can be detected using thermal or isothermal NAATs (nucleic acid amplification tests), sequencing, and hybridization, which are genotype-based diagnostic methods [63,64,65,66]. Second, microbial proteins can be detected by biochemical tests, rapid antigen tests, or MALDI-TOF MS (matrix-assisted laser desorption/ionization–time of flight mass spectrometry). Third, microorganisms themselves can be detected by cultures, a broad array of stain methods, and morphology-based methods, e.g., microscopy. Last but not least, host–protein biomarkers, cytokines/chemokines, antibodies, and CPE (cytopathogenic effect) during the host–microbe interactions can also be detected. In contrast to genotypic methods, the last three groups of testing methods are phenotype-based diagnostic methods. (**Right**) The pros and cons of the four groups of diagnostic methods are compared. CRP, C-reactive protein; IP-10, interferon gamma-induced protein 10; LAMP, loop-mediated isothermal amplification; NGS, next-generation sequencing; PCR, polymerase chain reaction; RT-PCR, real-time PCR; TRAIL, TNF-related apoptosis-inducing ligand; TMA, transcription-mediated amplification.

Historically, conventional procedures have been used for the identification of microorganisms to aid in the diagnosis of infectious diseases, which involves determining their traits, antigens, morphology, and human antibodies by performing cultures, microscopy, and serology (Figure 2, left) [56,57]. However, these diagnostic methods, based on phenotype, suffer from a delayed turnaround time (TAT), low sensitivity and specificity, and the requirement of special technical expertise (Figure 2, right). The paradigm of microorganism workup in the field of clinical microbiology has been dramatically changed with the advent of molecular biology and genotype-based diagnostic methods targeting nucleic acid (NA; DNA or RNA) [58,59]. For example, NA amplification (NAA) tests (NAATs), sequencing (Sanger or next-generation sequencing), and hybridization (Figure 2, left) have a short TAT, high sensitivity and specificity, and high accuracy compared with conventional phenotypic methods (Figure 2, right) [60,61,62].

The invention of multiplex PCR allows for the simultaneous detection of multiple organisms in one reaction [59]. However, there are several challenges associated with one-well multiplex PCR, including but not limited to undesirable primer–primer interactions, non-specific amplification, crosstalk between detection channels, and a limited number of detection channels [59]. Multiplex syndromic panels have been developed to overcome these hurdles, expand their panels to cover more organisms (Table 1). We discuss some examples of FDA-approved multiplex syndromic panels and their application in the detection of respiratory viruses [59]. Specifically, different vendors, for example, BioFire, GenMark (Roche), Qiagen, and Luminex, have different multiplex PCR panels, but they share some fundamental similarities, such as broad pathogen coverage, rapid turnaround time, and high sensitivity and specificity (Table 1).

### 3.1. BioFire Respiratory Panel 2.1

With the emergence of SARS-CoV-2, respiratory panels for diagnostic testing were altered to include SARS-CoV-2, one being the BioFire Respiratory Panel 2.1 [12]. The BioFire Respiratory Panel 2.1 (RP 2.1) is a multiplex PCR diagnostic tool that can detect singular infections and co-infections of 22 pathogens (19 viral and 3 bacterial) using nasopharyngeal swab specimens (Table 1) [67]. This highly efficient panel can be completed within approximately 45 min, which allows patients and physicians to determine the current course of the illness and treatment options more quickly. This test has overall 97.1% sensitivity and 99.3% specificity [16]. The BioFire Respiratory Panel 2.1 plus is an updated version that accounts for the original 22 pathogens, with the addition of MERS-CoV. This tool has an overall sensitivity of 97.1% and 99.3% specificity [17]. Considering that RP 2.1 and RP 2.1 plus both have high sensitivity and specificity, it can be inferred that they are efficient and accurate methods of detecting SARS-CoV-2 and other respiratory pathogens [66].

### 3.2. BioFire Spotfire

The BioFire Spotfire Respiratory Panel is a multiplex diagnostic tool that can detect up to 15 respiratory pathogens in approximately 15 min using nasopharyngeal or throat swab specimens (Table 1) [68]. It is typically used in point-of-care settings where rapid results are ideal for patient convenience. The overall sensitivity of BioFire Spotfire is 98.5%, while the specificity is 99.1%. For SARS-CoV-2 detection, the sensitivity is 97.3%, with a specificity of 99.4%. In addition, there is a smaller-scale version of this diagnostic test called the Spotfire Respiratory Panel Mini. This version can only target five pathogens, including SARS-CoV-2, human rhinovirus, influenza A, influenza B, and RSV. Furthermore, the overall sensitivity of this tool is 98.7%, while the specificity is 98%. For the detection of SARS-CoV-2, the sensitivity is 97.3%, with a specificity of 99.4% [14,19]. Both versions of this diagnostic tool have relatively high sensitivity and specificity, indicating that they provide accurate detection. However, in comparison to BioFire RP 2.1, they do not detect as many respiratory pathogens, so they cannot account for all possible co-infections. However, the turnaround time for this panel is far quicker (15 min vs. 45 min), which may be more feasible in POC settings, such as urgent care that need to supply patients with instant results.

### 3.3. Roche Cobas (GenMark) ePlex Respiratory Pathogen 2 (RP2) Panel

The Roche cobas ePlex RP2 Panel is a multiplex respiratory panel that can detect and differentiate nucleic acids from up to 17 viral and bacterial pathogens within 90 min using nasopharyngeal swap specimens (Table 1), with a sensitivity of 96–100% and a specificity of 96–100% (depending on various targets) [69]. Like the BioFire panels, for enterovirus and rhinovirus specifically, the cobas ePlex RP2 Panel cannot accurately differentiate between the two, so further testing would be needed for patients who test positive. This test was designed with the intention of lessening the occurrence of false positive results. However, false negatives and false positives are still possible outcomes, and patients should exercise caution if they believe they have been exposed regardless of their results [20,23]. The TAT of this panel, consisting of NA extraction, amplification, and detection, is 90 min, which is not as fast as the others. The TAT makes this panel more suitable for larger laboratory settings where rapid testing is not the priority.

### 3.4. Qiagen Respiratory Panel

The QIAstat-Dx Respiratory Panel Plus is a diagnostic respiratory panel that uses nasopharyngeal swab specimens to detect up to 21 viral and bacterial pathogens within an hour, with a sensitivity of 94–100% and a specificity of 97.9–100% (depending on various targets) [70,71]. This respiratory panel utilizes a multiplex RT-PCR that is intended for detecting co-infections in patient samples [72,73]. This is also referred to as syndromic testing, which means that it can quickly detect pathogens that have overlapping symptoms [74]. By being able to detect pathogens with similar symptoms, as well as co-infections, the QIAstat-Dx Respiratory Panel removes the need for multiple tests to diagnose patients [73]. In comparison to the other tests, it is better suited for diagnosing co-infections. Like the cobas ePlex respiratory panel, the QIAstat-Dx Respiratory Panel Plus cannot differentiate between enterovirus and rhinovirus. It has a slightly higher TAT than some of the other diagnostic tools at around 1 h, but the fact that it is a syndromic test is convenient in that it can eliminate the need for other diagnostic testing and is likely best used on patients experiencing symptoms that are common for multiple pathogens [66].

### 3.5. Luminex NxTAG Respiratory Pathogen Panel

The NxTAG Respiratory Pathogen Panel (NxTAG RPP) is a multiplex PCR diagnostic tool by Luminex (Table 1), with a sensitivity of 96–100% and a specificity of 96–100% (depending on various targets) [75,76]. This method of diagnostic testing can detect up to 19 pathogens of viral and bacterial origin, including SARS-CoV-2, from upper respiratory tract specimens. It takes around 3.5–5 h for the results to be available, but it can hold up to 48–96 patient samples at a time, as it houses 96-well plates. This makes it ideal in a setting like a hospital, where it may be most feasible to test patient specimens in large batches. Additionally, the main target for this method of testing is patients presenting with symptoms of respiratory tract infections [77,78]. In comparison with other respiratory diagnostic tools, the NxTAG RPP has a longer TAT for results, but it can test a larger number of patient specimens at once, which makes it more suitable in a larger-scale setting like a hospital or medical laboratory, where rapid testing in less than an hour is not the highest priority.

## 4. Conclusions

SARS-CoV-2 is a novel, highly-pathogenic HCoV that caused the COVID-19 pandemic around the world. The emergence of SARS-CoV-2 wiped out other respiratory viruses, e.g., influenza viruses and RSV, during the COVID pandemic, which is attributed to public health mitigation procedures. In the post-COVID era, the epidemiology and seasonality of traditional respiratory viruses has shifted partially due to their co-circulation with SARS-CoV-2. SARS-CoV-2 and traditional respiratory viruses share similar symptoms but may differ in clinical outcomes and treatment options, making the rapid detection and differentiation of SARS-CoV-2 and other traditional respiratory viruses critical. Multiplex molecular tests, e.g., PCR, are the main detection strategies. BioFire, GenMark, Luminex, and Qiagen technologies have their advantages and limitations, which are worth noting. This review provides updated information on the epidemiology, seasonality, and clinical significance of respiratory viruses and co-infections in the post-COVID era. It also sheds light on cutting-edge technologies for the simultaneous detection of respiratory viruses and SARS-CoV-2.

## Figures and Tables

**Table 1:** Comparison of commonly used multiplex PCR panels for the detection of respiratory viruses.

Technology	BioFire FilmArray	GenMark ePlex	Qiagen	Luminex
**NA amplification and detection methods**	Nested PCR, melt curve analysis	PCR, electrochemical detection	PCR	PCR, liquid phase bead array
**Turn-around time**	45 min (RP2.1); 15 min (Spotfire RP)	1.5 h	67–75 min	5 h
**Throughput**	1 test module/instrument; Torch: 2–12 test modules	3–24 per instrument	1 test module per instrument	96-well plate format
**Sensitivity**	97.1–98.5%	96–100%	94–100%	96–100%
**Specificity**	99.3–99.4%	96–100%	96–100%	96–100%
**Example of FDA-approved respiratory panels (number of targets)**	RP2 (22); BioFire Spotfire RP (up to 15)	RP (17)	QIAstat-Dx Respiratory Panel Plus (21)	x-TAG RVP (12); x-TAG RVP Fast (8); NxTAG RPP (20)

## Data Availability

Not applicable.
